# Experimental Data Extraction and in Silico Prediction of the Estrogenic Activity of Renewable Replacements for Bisphenol A

**DOI:** 10.3390/ijerph13070705

**Published:** 2016-07-12

**Authors:** Huixiao Hong, Benjamin G. Harvey, Giuseppe R. Palmese, Joseph F. Stanzione, Hui Wen Ng, Sugunadevi Sakkiah, Weida Tong, Joshua M. Sadler

**Affiliations:** 1 Division of Bioinformatics and Biostatistics, National Center for Toxicological Research, U.S. Food and Drug Administration, 3900 NCTR Road, Jefferson, AR 72079, USA; ng.huiwen@yahoo.com (H.W.N.); Suguna.Sakkiah@fda.hhs.gov (S.S.); Weida.Tong@fda.hhs.gov (W.T.); 2 Research Department, Chemistry Division, Naval Air Warfare Center Weapons Division, China Lake, Ridgecrest, CA 93555, USA; benjamin.g.harvey@navy.mil; 3 Department of Chemical and Biological Engineering, Drexel University, 3141 Chestnut St., Philadelphia, PA 19104, USA; grp27@drexel.edu; 4 Department of Chemical Engineering, Rowan University, Glassboro, NJ 08028, USA; stanzione@rowan.edu; 5 RDRL-WMM-C, Army Research Laboratory, 4600 Deer Creek Loop, Aberdeen Proving Ground, Aberdeen, MD 21005, USA

**Keywords:** bio-based, BPA replacement, endocrine disruption ER

## Abstract

Bisphenol A (BPA) is a ubiquitous compound used in polymer manufacturing for a wide array of applications; however, increasing evidence has shown that BPA causes significant endocrine disruption and this has raised public concerns over safety and exposure limits. The use of renewable materials as polymer feedstocks provides an opportunity to develop replacement compounds for BPA that are sustainable and exhibit unique properties due to their diverse structures. As new bio-based materials are developed and tested, it is important to consider the impacts of both monomers and polymers on human health. Molecular docking simulations using the Estrogenic Activity Database in conjunction with the decision forest were performed as part of a two-tier in silico model to predict the activity of 29 bio-based platform chemicals in the estrogen receptor-α (ERα). Fifteen of the candidates were predicted as ER binders and fifteen as non-binders. Gaining insight into the estrogenic activity of the bio-based BPA replacements aids in the sustainable development of new polymeric materials.

## 1. Introduction

Bisphenol A (BPA, 4,4′-(propane-2,2-diyl)diphenol) is one of the most ubiquitous molecules in modern polymer science [1]. Since its commercialization in 1950, BPA can be found in thermoplastic and thermosetting resins including vinyl esters, higher performance epoxy resins, and polycarbonates [1]. Production of BPA exceeds six billion tons a year, with annual consumption increasing at >5% a year [2,3]. BPA is used in a wide array of products including food and beverage containers, water piping, safety equipment, coatings, adhesives, and high performance composites as well as automotive and aircraft parts [2,3]. Due to the wide spread use of BPA and the expectation of prolonged exposure [4,5,6], significant public scrutiny has emerged regarding the health effects of continued exposure to BPA [6,7]. Reports from the early 2000s state that the majority of the U.S. population display detectable levels of BPA in their urine, which has led to increased public scrutiny [7].

Concerns over BPA have grown in the last decade due to reports of its putative endocrine-interfering nature and potential to cause a myriad of adverse health effects. BPA has been shown to bind to a variety of receptors in biological systems, such as the estrogen receptor-α (ERα), androgen receptor, and thyroid hormone receptor [8]. The ability of BPA to mimic the endogenous estrogens and act as a weak xenoestrogen [9] through binding to the notoriously promiscuous ERs [10] has led to an extensive study of BPA. Animal studies showed that at high doses, BPA produced estrogen-like effects, e.g., increased uterine and prostate weight [7]. At the lowest adverse effect level (LOAEL) of 50 mg (kg body weight)^−1^ day^−1^ [6], effects on the mammary glands [11] and urogenital system [12], including morphological changes in testes and sperm [13], were observed. The latter effect was proposed to be a product of BPA binding to membrane-bound ERs [14]. Ultimately, the mechanism of BPA endocrine activity remains unclear, and although the safety of BPA currently remains contentious, it is generally considered safe at the current levels and is continuing to be approved for uses in food containers and packaging by the U.S. Food and Drug Administration (FDA) [15]. However, some countries (e.g., European Commission [16] and the Government of Canada [17]) have enacted laws to ban BPA from certain products, including milk bottles for infants.

Unfortunately, the widespread use of BPA cannot be mitigated by simply eliminating its usage. BPA is the core scaffold for many polymer systems, and it is the critical structural feature from which many polymers derive their properties and performance. Investigations into alternative bisphenols have displayed limited success, but ultimately have not been able to meet the performance standards including stiffness, modulus and glass transition temperature (Tg) exhibited by BPA based polymers. Although substantial modification of the core structure may be possible, structure property relationship studies suggest that replacement monomers will need to be bifunctional and contain rigid ring structures [18]. New compounds that feature two or more sites for functionalization are critical in the development of BPA replacements to insure the participation of the molecules in polycondensation reactions that result in thermoplastic materials, such as polyurethanes and polycarbonates, which are utilized for coatings, food packaging, and safety equipment. The bifunctional nature of BPA is also necessary for the development of new cross-linking monomers used in thermosetting resins found in high performance epoxy, cyanate ester, and vinyl ester resins. The ring structures in the core scaffolding of BPA have been shown to increase thermomechanical properties of polymerized materials [18]. Backbone rings (thermoplastics) and rings in cross-linked chains (thermosets) increase the stiffness, strength and Tg of polymers and result in the performance characteristics that have become a hallmark of BPA [19]. New BPA replacements should emulate these features in order to drive properties of novel, sustainable polymers toward operating windows that can compete with BPA based polymers.

In recent years, biorefineries have emerged that produce a wide array of renewable building blocks useful as platform chemicals for the development of polymers, pharmaceuticals and other commercially significant products [20]. The use of bio-derived materials as precursors to polymers is a field of increasing interest due to the potential to reduce environmental impact, increase global sustainability, produce molecules with reduced toxicity, and reduce production costs [21,22]. Modern bio-refining processes use a number of different feedstock streams, such as cellulose, carbohydrates, plant oils, lignin, tanninoids, and terpenoids, that can be used to produce novel platform chemicals [20] with structural features that are not readily available from traditional petrochemical refining processes. With the development of the appropriate chemistries to utilize their structural features, these platform chemicals can be used as precursors to new monomers that have the potential to be phenolic replacements with both improved performance and reduced toxicity.

There are a number of instances in the literature where novel bio-based monomeric materials have been used to develop renewable thermoplastic and thermosetting resins. Carbohydrate derived molecules, including isosorbide and bishydroxymethylfuran (BHMF), have been incorporated into polyesters, vinyl esters, and epoxy resins; some of these materials display high performance characteristics with the potential to compete with BPA based materials [23]. Guaiacyl structures are lignin derived molecules that can be converted to substituted variants of BPA [23]. In many cases, these variants have material properties that are directly comparable to BPA and have been incorporated into vinyl ester and epoxy resins systems. Additionally, these renewable lignin derived molecules have been incorporated into a polycarbonate that shows competitive properties [23]. Recent work has shown that the lignin-derived phenols, creosol [24,25,26], and vanillin [27], can be efficiently converted to bisphenolic and dianiline derivatives to produce high temperature thermosetting resins and polycarbonates. Similarly, eugenol [28,29], anethole [30], resveratrol [31,32] and even p-cymene (a component of pine resin) [33,34] have been used as starting materials for the synthesis of high performance polymers. Additionally, ferulic acid-based bisphenols materials have shown promise as potential BPA replacements for use in epoxy resins and show reduced estrogenic activity [35,36].

Despite the promise of new bio-based polymers and thermosetting resins, there is relatively little known about the potential biological activity of these sustainable materials. For any new compound (bio-based or not) to become a viable replacement for bisphenol A or methylene dianiline, the candidates must go through a rigorous risk assessment. As endocrine disruption potential through ER mediated responses is one of the safety concerns of replacement compounds, prediction of estrogenic activity of potential alternatives can prioritize candidates to obtain experimental measurements of their estrogenic activity.

To analyze estrogenic activity of the potential replacement molecules, we searched experimental data using publically available databases. Most of the compounds do not have experimental data available. We then used a consensus modeling approach to predict estrogenic binding activity of the molecules. The consensus model consists of two individual models that were developed using two different training datasets. The two individual models were validated through five-fold cross validations and an external validation. Model results showed half of the compounds were predicted to be potential ER binders. The binding affinities of the predicted ER binders were further estimated using a quantitative prediction model. Several of the predicted compounds displayed similar estrogenic activity to BPA, however there were a number of candidates that were calculated as non-binders. These results demonstrate that further risk assessment of the potential BPA replacement compounds is needed and show that current work offers potential molecules that possess reduced activity or no activity that would satisfy the public’s concern for the safety of products using the replacement molecules as alternatives to BPA.

## 2. Materials and Methods

### 2.1. Study Design

**Figure 1:**
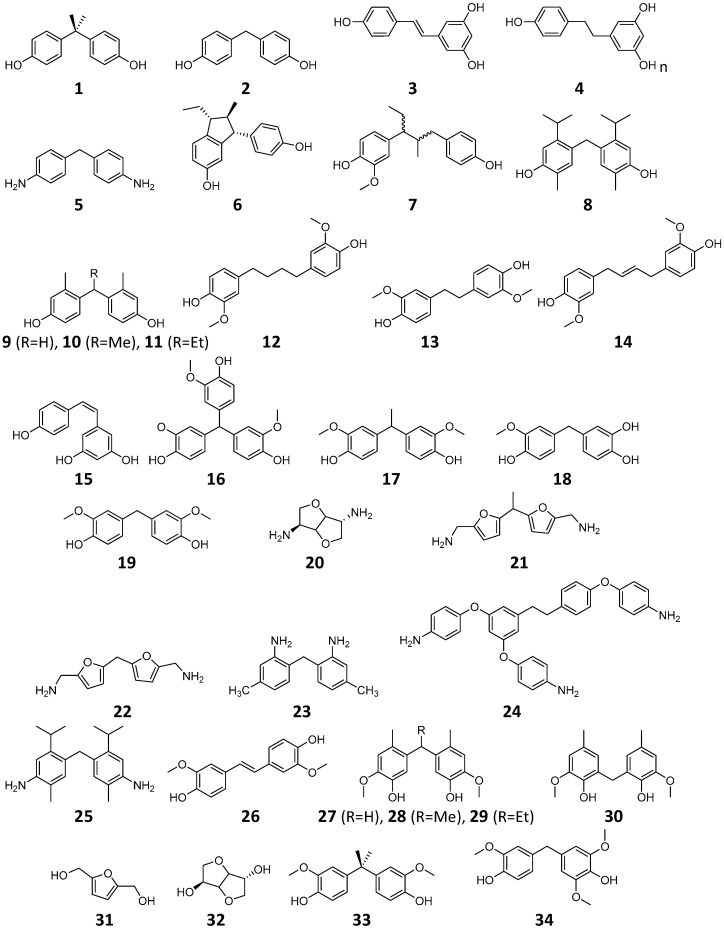
Structures of the 34 potential BPA replacement compounds and the reference compound BPA. The numbers under structures were used in the text and Tables.

For the 34 potential replacement compounds shown in Figure 1, the estrogenic activity was analyzed by extracting experimental data and in silico predictions. The reference compound, BPA, and a small subset of the 34 potential replacement molecules for which experimental data were available, were obtained through searching the estrogenic activity database (EADB) [37]; our recently updated database in the endocrine disruptors knowledge base (EDKB) [38].

**Figure 2:**
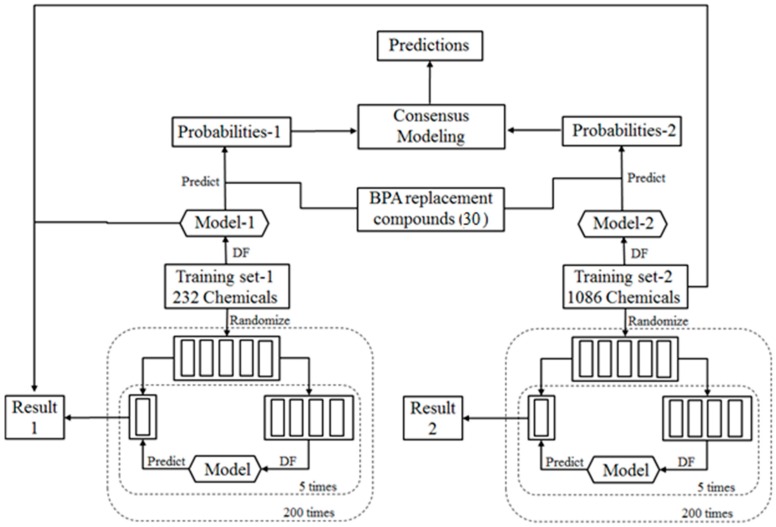
Workflow of the qualitative consensus modeling for prediction of compounds as estrogen receptor (ER) binders and non-binders.

For the potential BPA replacement compounds that had no estrogenic data in EADB, estrogenic activity was predicted in two tiers. In the first tier, all compounds were qualitatively predicted to be either an ER binder or non-binder using the workflow that is detailed in Figure 2. In the second tier, ER binding affinities of the potential BPA replacement compounds that were predicted to be ER binders were estimated using a quantitative prediction model developed in our previous study [39].

The overall workflow for qualitative prediction model development is illustrated in Figure 2. The in-house experimental ER binding activity data of 232 chemicals were used as a training data set (TS-1) to construct an individual prediction model; our previously curated estrogenic activity data of 1086 chemicals were used as another training data set (TS-2) to build another individual model. To demonstrate that reliable individual models can be developed from the training data sets, 200 iterations of five-fold cross validations were conducted on both training data sets. Most of the chemicals in TS-2 were not included in TS-1 and were used as an external data set to challenge the prediction model constructed using TS-1 for further evaluation of the performance of the individual models. For the 29 potential BPA replacement compounds for which no experimental data was available, two individual models (M-1 and M-2) were used to predict the probabilities of these compounds to be ER binders. The two sets of probabilities were then averaged to make consensus predictions on the compounds.

### 2.2. Training Data Sets

Two training data sets were used. Both data sets contained the ER binding activity measured using the competitive rat ER binding assay in our previous studies [40] and can be obtained from our databases EADB and EDKB [37,38]. Training set 1 (TS-1) contained 232 chemicals, of which 131 showed ER binding activity and were defined as ER binders. The remaining 101 structures did not show ER binding activity and were defined as ER non-binders. TS-1 has been used for the development of ER binding activity prediction models [41,42,43]. Training set 2 (TS-2) contained 1086 chemicals whose estrogenic activity data were curated from the literature. From these structures, 350 of the 1086 were categorized as ER binders with the remaining 736 designated as ER non-binders [44].

### 2.3. BPA Replacement Compounds

Most of the molecules seen in Figure 1 are potential bisphenolic and dianiline replacements that have been synthesized from bio-based building blocks. A number of these compounds are produced from platform chemicals that can be derived from bio-refined carbohydrate feedstocks (**20**–**22**, **31** and **32**) and have shown potential as precursors to resins that can be replacements for BPA based epoxy and vinyl ester resins [45,46,47]. Lignin derived compounds are also an area of interest due to the high phenolic content of lignin. Of the structures shown in Figure 1, 17 are constructed from lignin model compounds (**9**–**14**, **16**–**19**, **26**–**30**, **33** and **34**) and have shown promising results in the development of new thermosetting monomers and thermoplastics that may serve as direct replacements for BPA-derived polymers [24,25,26,27,48,49]. Compounds **3**, **4**, **15**, and **24** are based on resveratrol, a stilbene that can be isolated from plants, generated from sugar by fermentation with metabolically engineered yeast, or generated by hybrid fermentation/chemical synthesis routes. Compounds **6** and **7** are derived from anethole [30], an aromatic compound present in pine resin and various essential oils. Compounds **8** and **25** can be derived from terpenes present in both pine resin and citrus peels [33,34]. The remaining compounds (**1**, **2**, **5**, and **23**) are petroleum derived bisphenols and anilines included for comparison.

### 2.4. Molecular Descriptors

Molecular descriptors were calculated for the molecules in TS-1 and TS-2 as well as the 34 potential BPA replacement compounds and the reference compound BPA using our previously developed molecular descriptors calculation software Mold2 [50,51]. Mold2 is a freely available software package that calculates molecular descriptors from two-dimensional chemical structures and has been demonstrated to be a reliable tool for QSAR models development [52,53,54,55]. One of the attractive features of this software package is its high computational speed due to adoption of an extremely fast algorithm for ring structure recognition [56] and the utilization of an efficient representation system for chemical structures [57,58] that have been developed in the structure elucidation system based on nuclear magnetic resonance (NMR) [59,60,61] and infrared (FT-IR) spectra [62]. For each structure, 777 molecular descriptors were calculated from Mold2 and then preprocessed by removing duplicate values for all the candidates in TS-1 or TS-2. The remaining descriptors were used in the development of the models.

### 2.5. Individual Models

Decision Forest (DF) is a machine learning algorithm that incorporates numerous decision tree models to make accurate predictions [63,64,65]. Incorporating multiple identical decision tree models is unlikely to improve prediction accuracy. Instead, the DF algorithm uses decision tree models that have good performance and are constructed using different chemical features to make a final prediction. The chemical diversity for each decision tree model within the DF warrants that each member model contributes to the prediction in different but complementary ways; the algorithmic description of the DF can be found in our previous publications [63,64,65]. Briefly, DF modeling has four components: (1) develop a decision tree using all molecular descriptors; (2) remove the descriptors that have been used in the previous decision tree; (3) iterate Steps (1) and (2) until the improvement in training is no longer improved by adding additional decision trees; and (4) predict the activity of a candidate by combining the results of all the decision trees.

In each member tree model, the probability for assigning a structure to be active is a value between 0 and 1; this value is computed from the number of active molecules in the terminal node and the size of the node. The average probability value across all the member tree models for a molecule is used to predict the activity of a given molecule. If the average probability is greater than 0.5, the structure is determined to be active and inactive if the probability is less than or equal to 0.5.

### 2.6. Prediction Performance

The prediction results of DF models can be measured using different metrics. We used prediction accuracy, sensitivity, specificity, Matthews correlation coefficient (MCC) and balanced accuracy to measure our models. These performance metrics were calculated using Equations (1)–(5) for comparison of the predictions with the actual activity classes (binder and non-binder).

(1) $$Accuracy=\frac{TP+TN}{TP+TN+FP+FN}$$

(2) $$Sensitivity=\frac{TP}{TP+FN}$$

(3) $$Specificity=\frac{TN}{TN+FP}$$

(4) $$MCC=\frac{TP\times TN-FP\times FN}{\sqrt{\left(TP+FP\right)\left(TP+FN\right)\left(TN+FP\right)\left(TN+FN\right)}}$$

(5) $$Balanced\;Accuracy=\frac{TP\left(TN+FP\right)+TN\left(TP+FN\right)}{2\left(TP+FN\right)\left(TN+FP\right)}$$

In Equations (1)–(5), true positive (TP) is the number of ER binders that were predicted as ER binders by the DF models, true negative (TN) is the number of ER non-binders that were predicted as ER non-binders, false negative (FN) is the number of ER binders that were predicted as ER non-binders, and false positive (FP) is the number of ER non-binders that were predicted as ER binders.

### 2.7. Cross Validations

To evaluate the performance of DF models, we used 5-fold cross validations for both TS-1 and TS-2 as shown in Figure 2. In one 5-fold cross validation, the structures in the training data set (TS-1 and TS-2) were randomly split into five equal portions. Four of the five portions were used to construct a DF model, before using the model to predict ER binding activity for the remaining portion. This process was repeated sequentially so that each of the five portions was left out once and only once as the testing set. The prediction results from the 5 DF models were then averaged to provide an estimate of model performance. The 5-fold cross-validation was iterated 200 times using different random divisions of the structures into 5 equal portions to reach a statistically robust estimation for the DF model performance.

### 2.8. External Validations

QSAR models in cross validations usually perform better than models tested on a new data set. Validation using external data sets is more reliable and necessary to evaluate the performance of a QSAR model. In this study, TS-2 contained 1086 molecules with known ER binding activity data. Most of the molecules were not included in TS-1 and were used as an external validation data set to test Model-1 that was constructed using the 232 molecules in TS-1 as shown in Figure 2.

### 2.9. Consensus Modeling

QSAR models have limitations due to diverse reasons such as limitation of the chemical structural space covered by the training molecules. To improve prediction accuracy, a consensus strategy was utilized to predict estrogenic activity of the potential BPA replacement molecules. The estrogenic activity of each molecule was predicted separately using the DF models that were constructed using TS-1 and TS-2. DF classification models output the probabilities of the molecules likely to be ER binders. The two probabilities were then averaged to make a consensus prediction of estrogenic activity (ER binder or non-binder) for the structure.

### 2.10. Prediction Confidence

The ER binding activity (binder: *p* > 0.5 or non-binder: *p* ≤ 0.5) prediction from the consensus modeling of the DF models for a molecule is a continuous probability. This value represents the confidence for the prediction. A good prediction is expected to have a high confidence level. The prediction confidence was calculated for each of the predictions from the consensus modeling of the DF models using Equation (6).

(6) $$Confidence=\frac{\left|\left(p-0.5\right)\right|}{0.5}$$

The calculated prediction confidence is a value between 0 and 1. The larger the value is, the more reliable is the prediction.

### 2.11. Quantitative Prediction

Quantitative predictions were conducted on the potential BPA replacement molecules that were predicted to be ER binders based on the qualitative predictions derived from the in silico molecular docking model developed in our previous study [34]. The model uses binding free energies of the BPA replacement molecules that were predicted to be ER binders based on molecular docking them into an ER ligand binding pocket. Molecular docking was conducted with the Glide Extra Precision scoring function in Maestro [66] with a grid box of 20 by 20 Å. The parameters used in this docking analysis were flexible ligand sampling, a 2.5 kcal/mol energy window for ring sampling, 5000 poses per ligand at the initial phase of docking, 400 poses per ligand for energy minimization, and a maximum of 100 minimization steps. For each structure, five poses were used for the post-docking minimization and the best pose was outputted for binding free energy calculations. The binding free energies of the predicted estrogenic BPA replacement molecules were calculated using Prime MM-GBSA v3.6 within Maestro. The residues within 3.0 Å of the docked BPA replacement compound were allowed to be flexible and the rest of the parameters were set to the default in the calculations. The binding free energies of the predicted estrogenic BPA replacement compounds were converted to logarithmic relative binding affinity (log RBA) values using the linear regression model presented in Equation (7) [39].

(7) logRBA=−7.719−0.086×(binding free energy)

## 3. Results

### 3.1. Experimental Estrogenic Data

Of the 34 potential BPA replacement molecules searched in EADB, four were included in the database. The experimental estrogenic data curated in EADB for these four compounds as well as the reference compound BPA are given in Table 1. Compound **4** showed no activity in the cell proliferation assay [67] and compound **5** was experimentally determined to be an ER non-binder [38]. Compounds **2** and **3** showed weak estrogenic activity in experiments compared with the reference compound, BPA (compound **1**) [38,68]. The experimental ER binding affinities are given in Table 1 as logRBA values normalized to the natural hormone estradiol with a logRBA set to 2.

### 3.2. Cross Validations

**Figure 3.**
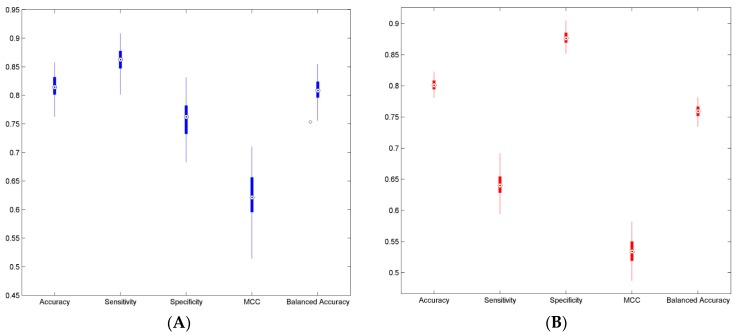
Boxplot of performance parameters of the five-fold cross validations. Prediction accuracy, sensitivity, specificity, Mathews correlation coefficient (MCC) and balanced accuracy of the 200 iterations of five-fold cross validations were plotted for TS-1 (**A**); and TS-2 (**B**). The parameters were indicated at the x-axis and their values were presented in the y-axis.

To qualitatively predict ER binding activity of the potential BPA replacement molecules, we constructed classification models using two training data sets. The five-fold cross-validations were carried out on the two training data sets as internal validations for estimation of the performance of each prediction model. The five performance parameters were calculated using Equations (1)–(5) for the 200 iterations of five-fold cross validations and were plotted as box plots for TS-1 (Figure 3A) and TS-2 (Figure 3B). The mean values and standard deviations of the five performance parameters for the two training sets are given in Table 2 as Result-1 and Result-2 columns, respectively. The overall prediction accuracy of >80% demonstrates good prediction models could be constructed from the two training data sets. The small standard deviations for all performance parameters indicate that the models performed consistently and, thus, the prediction models constructed from the two training data sets should be statistically reliable.

### 3.3. External Validations

Because most of the structures in TS-2 are not included in TS-1, those structures were used for external validations of the prediction model constructed from TS-1. A DF prediction model, Model-1, was first constructed using all of TS-1. Model-1 was then used to predict the external validation structures from TS-2. The predictions had the following parameters: prediction accuracy, 0.770; sensitivity, 0.803; specificity, 0.754; MCC, 0.527 and balanced accuracy, 0.778. Comparing the five-fold cross validation results shown in Table 2 reveals that the external validations had similar performances, though they slightly underperformed the cross validations, further indicating that reliable predictions could be achieved with the DF models constructed from the training data sets.

### 3.4. Qualitative Predictions

We used consensus modeling to qualitatively predict estrogenic activity (classify structures as either ER binders or non-binders) for the 29 potential BPA replacement molecules without experimental estrogenic activity data available. As shown in Figure 2, qualitative prediction models, Model-1 and Model-2, were first constructed using the DF algorithm based on TS-1 and TS-2, respectively. The models were then applied to assess the probabilities that each of the 29 molecules would act as ER binders. The two probabilities calculated for each molecule were then averaged to obtain a consensus evaluation of the predicted ER binding affinity for a given structure. The results of the consensus modeling on the 29 potential BPA replacement molecules are given in Table 1. Of the 29 compounds, 14 were predicted as ER binders by the consensus model while the remaining 15 were predicted as ER non-binders.

The probability for a compound to be an ER binder from the consensus modeling was converted to prediction confidence using Equation (6). The prediction confidence values of the 29 molecules are listed in Table 1. Most of the predictions, especially for the 14 predicted ER binders, had reasonably high confidence levels of greater than 0.6 that means probability of the chemical in prediction to be an ER binder is higher than 80%, indicating that the predictions can be considered reliable based on the modeling parameters used.

### 3.5. Quantitative Predictions

For the 14 potential BPA replacement compounds that were predicted to be ER binders, their binding affinities were further estimated using the quantitative prediction model developed in our previous study [39]. The quantitative predictions are given in the last column of Table 1. Most of these 14 compounds were predicted as weak ER binders with estimated binding affinities similar to the experimentally determined binding affinity for the reference compound, BPA. Only compound **8** showed more than 100 fold higher ER binding affinity than BPA; but the consensus qualitative DF model had a low prediction confidence level. Compound **16** has a relatively large size and its molecular shape could not fit into the ER ligand binding pocket that was adopted from an ER complex bound with an ER agonist in the protein data bank. It failed in the docking and, thus, its ER binding affinity could not be estimated using this quantitative prediction model. Therefore, compound **16** is likely a non-binder based on the docking model though it was predicted to an ER binder at low confidence 0.584.

## 4. Discussion

Experimentally, the natural agonist of the E2, 17β-estradiol, has a log RBA value of 2.0, and a calculated log RBA of 0.226 [39]. Compounds that have been experimentally determined to show activity in the E2 receptor, such as BPA and bisphenol-F (BPF), display log RBA values of −1.680 and −3.050, respectively [39], indicating that when compared to the parent substrate, these compounds show significantly less affinity to E2. However, they still possess enough structural features to be recognized and activate the receptor. When the logRBA is calculated in silico for BPA and BPF (−1.895 and −3.158, respectively) [39], the values are slightly reduced from the experimentally calculated values, however, the reasonable accuracy of the model suggests that it is a useful tool to inform the development of new monomers. Replacement candidates should show calculated log RBA values that are significantly lower than that of the natural agonist. In this instance, values lower than −3.2 would outperform compounds that are already known activators; ideally, the qualitative prediction would show the compounds as non-binders and would yield no discernable log RBA value. Of the 29 candidate structures studied, many (15 compounds) resulted in qualitative predictions that indicated “non-binder” with the remaining 14 structures categorized as potentially active. The calculated log RBA values should be helpful for the development of new monomers and refining of the model.

Compounds that were calculated to be non-binders possessed a broad array of structural features that could prevent recognition in the E2 receptor. Furans and bicyclic rings used in place of the bisphenolic rings seemed to have a significant effect on recognition as well as structures where the phenolic hydroxyl was located in a position other than the common orientation para to the bridging group. Among the group of “probable inactive” compounds were structures bearing amine groups; aniline derivatives show no recognition using molecular simulations and literature SAR studies have shown that amine modification on the aromatic ring diminishes binding affinity and reduces molecular recognition [69].

Another factor to consider is the ability of a candidate structure to adopt a planar configuration. Estradiol and its active metabolites consist of a series of rings that separate the terminal hydroxyl groups. The hydroxyl groups are separated by nine carbons that position them for recognition at opposite ends within the E2 receptor [69]. BPA and its derivatives fulfill these basis structural characteristics for recognition in the E2 receptor; the potential replacements (**6**–**19**) were similar enough to the base bisphenolic structure to be used in the molecular calculations to determine the relative binding affinity in silico. These molecules were structurally similar to that of BPA, exhibiting two aromatic rings separated by aliphatic bridges with various shapes and sizes. The structures where the aromatic rings separated by a single carbon unit (methylene, ethylene, isopropylidene) seem to have a minimal effect on the calculated log RBA. Changing the spacer to an ethyl or vinylic group seemed to have a more profound effect on the calculated binding affinity of the potential replacements, resveratrol (**3**) displayed a log RBA of −2.489, considerably better than that of BPA; however, dihydro-resveratrol was calculated to be inactive by the DF. By reducing the double bond that separates the aromatic rings, it would appear that it is more difficult for the structure to adopt the planar conformation needed for recognition; however, increasing the spacer to C > 3 (**6**, **7**, **12**, and **14**) seemed to allow the structure to adopt conformation that would allow for recognition.

Many potential replacements for BPA used in this investigation were similar in structure, with only minor variations around the aromatic rings. Compounds that were functionalized with aliphatic groups (**8**–**11**) seemed to perform poorly in the molecular calculations resulting in log RBA values (0.786 to −1.903) that were similar or greater than that of the BPA. Compound **8**, a modified bisphenolic with a methyl and isopropyl group on each aromatic ring, proved to be the poorest performer of the analyzed structures with a log RBA of +0.786. The literature confirms that the addition of methyl groups on the aromatic ring has a minimal effect on the binding affinity; however, the addition of multiple and larger aliphatic groups has the potential to increase the binding affinity to the E2 receptor [69]. Conversely, the same investigation showed that the addition of methoxy groups around the aromatic ring significantly decreased binding affinity [69]. Molecular calculations for methoxy substituted phenolic derivatives showed similar log RBA values to that of BPA with compounds **33** and **34** calculated to be non-binding.

## 5. Conclusions

The DF was able to identify 15 of the 29 potential replacement compounds as non-binders, however many of the structures that were calculated to be “active” in ER showed reduced log RBA values when compared to BPA. The structural modification of the potential replacements displayed calculated effects that were congruent with literature QSAR studies on the binding affinity of agonist metabolites. These results are promising for the development of renewable replacement molecules for resins that utilize diphenyl methane as the core structural feature. Many of the compounds studied in this work offer alternatives to BPA that are potentially lower in ER activity and can be used as components of novel materials that have similar thermomechanical properties compared to materials prepared from BPA. However, the confidence values for the compounds of interest were well below 50% for many of the studied molecules. This indicates that further studies are necessary to optimize the results. ER binding assays with the replacement compounds will give a clearer picture of the binding activity of the molecules and may provide further information to hone the DF calculation to give more accurate prediction for subsequent studies on potential replacements.

## Figures and Tables

**Table 1:** Estrogenic activity predictions of potential BPA replacement compounds.

Compound		Qualitative Prediction *				Quantitative Prediction **
#	Name	TS-1	TS-2	Predict	Confidence	
**1**	Bisphenol-A (BPA)					−1.532 (±0.664, *n* = 21)
**2**	Bisphenol-F (BPF)					−2.544 (±1.232, *n* = 3)
**3**	Resveratrol					−2.489 (±0.016, *n* = 2)
**4**						
**5**	MDA					
**6**		0.984	1.000	+	0.984	−1.004
**7**		0.984	0.800	+	0.784	−0.831
**8**		0.788	0.600	+	0.388	0.786
**9**		0.984	1.000	+	0.984	−0.704
**10**		0.984	1.000	+	0.984	−1.903
**11**		0.984	1.000	+	0.984	−1.064
**12**		0.984	0.943	+	0.927	−0.380
**13**		0.784	0.543	+	0.327	−2.338
**14**		0.784	0.743	+	0.527	−0.214
**15**		0.984	0.972	+	0.962	−2.222
**16**	Triguaiacol	0.984	0.600	+	0.584	NA
**17**	Bisguaiacol E	0.834	0.600	+	0.434	−1.117
**18**	BGF-Catechol	0.984	0.943	+	0.927	−1.862
**19**	Bisguaiacol-F (BGF)	0.984	0.600	+	0.584	−1.760
**20**	MDA-13	0.003	0.004	−	0.993	
**21**	Me-DFDA	0.003	0.404	−	0.592	
**22**	DFDA	0.203	0.404	−	0.392	
**23**	MDA-30	0.123	0.401	−	0.475	
**24**	MDA-13	0.317	0.444	−	0.238	
**25**	p-Cymene	0.453	0.333	−	0.213	
**26**		0.216	0.400	−	0.384	
**27**		0.616	0.300	−	0.084	
**28**		0.316	0.350	−	0.334	
**29**		0.566	0.300	−	0.134	
**30**		0.566	0.300	−	0.134	
**31**	BHMF	0.033	0.363	−	0.604	
**32**	Isosorbide	0.203	0.363	−	0.434	
**33**	Bisguaiacol A	0.516	0.250	−	0.234	
**34**	BGF-Syringol	0.366	0.400	−	0.234	

* Qualitative prediction: columns “TS-1” and “TS-2” give the probabilities of chemicals predicted as ER binders from the models trained on TS-1 and TS-2; column “Predict” gives the consensus qualitative prediction: the “+” indicates ER binder and “−” mean non-binder; ** Numbers are in logRBA; “NA” indicates that quantitative prediction was conducted but failed to predict; empty cells means quantitative predictions were not conducted as they were predicted as non-binders; for compounds **1** to **3** data are from multiple experiments (n indicates number of experiments), average values given in the parentheses, numbers after “±” in the parentheses are standard deviations; experiments showed no activity for **4** and **5**.

**Table 2:** Cross validation results.

Parameter	Result-1 (Mean ± Std)	Result-2 (Mean ± Std)
Accuracy	0.812 (±0.019)	0.801 (±0.009)
Sensitivity	0.861 (±0.020)	0.641 (±0.018)
Specificity	0.758 (±0.033)	0.877 (±0.011)
MCC	0.624 (±0.039)	0.534 (±0.021)
Balanced Accuracy	0.809 (±0.020)	0.759 (±0.010)

Std: standard deviation; MCC: Mathews correlation coefficient.
